# Gray Matter Network Disruptions and Regional Amyloid Beta in Cognitively Normal Adults

**DOI:** 10.3389/fnagi.2018.00067

**Published:** 2018-03-15

**Authors:** Mara ten Kate, Pieter Jelle Visser, Hovagim Bakardjian, Frederik Barkhof, Sietske A. M. Sikkes, Wiesje M. van der Flier, Philip Scheltens, Harald Hampel, Marie-Odile Habert, Bruno Dubois, Betty M. Tijms

**Affiliations:** ^1^Alzheimer Center & Department of Neurology, Amsterdam Neuroscience, VU University Medical Center, Amsterdam, Netherlands; ^2^Department of Psychiatry & Neuropsychology, School for Mental Health and Neuroscience, Maastricht University, Maastricht, Netherlands; ^3^Département de Neurologie, Pitié-Salpêtrière University Hospital, Institut de la Mémoire et de la Maladie d’Alzheimer, Paris, France; ^4^Institut du Cerveau et la Moelle Epinière (ICM)/Brain and Spine Institute, Pitié-Salpêtrière Hospital, Sorbonne Universities, Pierre and Marie Curie University, Paris, France; ^5^Department of Radiology and Nuclear Medicine, Amsterdam Neuroscience, VU University Medical Center, Amsterdam, Netherlands; ^6^Institutes of Neurology and Healthcare Engineering, University College London, London, United Kingdom; ^7^Department of Epidemiology and Biostatistics, VU University Medical Center, Amsterdam, Netherlands; ^8^AXA Research Fund & Sorbonne University Chair, Paris, France; ^9^Sorbonne University, GRC no. 21, Alzheimer Precision Medicine (APM), AP-HP, Pitié-Salpêtrière Hospital, Paris, France; ^10^Nuclear Medicine Department, Laboratoire d’Imagerie Biomédicale, Sorbonne Universités, Pitié-Salpêtrière University Hospital, Paris, France

**Keywords:** amyloid beta, PET, gray matter network, graph theory, MRI, subjective memory complaints, Alzheimer’s disease

## Abstract

The accumulation of amyloid plaques is one of the earliest pathological changes in Alzheimer’s disease (AD) and may occur 20 years before the onset of symptoms. Examining associations between amyloid pathology and other early brain changes is critical for understanding the pathophysiological underpinnings of AD. Alterations in gray matter networks might already start at early preclinical stages of AD. In this study, we examined the regional relationship between amyloid aggregation measured with positron emission tomography (PET) and gray matter network measures in elderly subjects with subjective memory complaints. Single-subject gray matter networks were extracted from T1-weigthed structural MRI in cognitively normal subjects (*n* = 318, mean age 76.1 ± 3.5, 64% female, 28% amyloid positive). Degree, clustering, path length and small world properties were computed. Global and regional amyloid load was determined using [^18^F]-Florbetapir PET. Associations between standardized uptake value ratio (SUVr) values and network measures were examined using linear regression models. We found that higher global SUVr was associated with lower clustering (*β* = −0.12, *p* < 0.05), and small world values (*β* = −0.16, *p* < 0.01). Associations were most prominent in orbito- and dorsolateral frontal and parieto-occipital regions. Local SUVr values showed less anatomical variability and did not convey additional information beyond global amyloid burden. In conclusion, we found that in cognitively normal elderly subjects, increased global amyloid pathology is associated with alterations in gray matter networks that are indicative of incipient network breakdown towards AD dementia.

## Introduction

Amyloid pathology is hypothesized to be one of the earliest events in the pathological cascade of Alzheimer’s disease (AD; Jack et al., 2013; Villemagne et al., 2013), and has been associated with future cognitive decline in cognitively normal subjects (Donohue et al., 2017). Understanding associations between amyloid pathology and other early pathological processes is critical as secondary prevention trials are shifting towards the earliest disease stages. AD can be considered as a disconnectivity disease (Delbeuck et al., 2003). In this study, we examined the relation between amyloid depositions measured with positron emission tomography (PET) and disruptions of gray matter networks in elderly subjects.

Brain areas involved in similar cognitive functions tend to develop in a coordinated way (Andrews et al., 1997; Alexander-Bloch et al., 2013b; Váša et al., 2018). Such co-variation of gray matter structure can be measured using structural T1-weighted MRI images and represented as a network (Lerch et al., 2006; Bassett et al., 2008; Tijms et al., 2012; Alexander-Bloch et al., 2013a). In cognitively normal subjects, brain networks tend to have a “small-world” organization, and it has been proposed that such a network organization provides an optimal balance of specialized information processing and integration (Sporns et al., 2004; Humphries and Gurney, 2008; Alexander-Bloch et al., 2013a). Using group level approaches (i.e., one network per diagnostic group), several studies have shown that gray matter network measures are disrupted in AD dementia compared to controls (He et al., 2008; Yao et al., 2010; Pereira et al., 2016). Using our method to extract gray matter networks on a single-subject level (Tijms et al., 2012), we have shown that worse gray matter network disruptions in AD dementia are associated with more severe symptoms, and worse functioning in specific cognitive domains (Tijms et al., 2013a, 2014).

In cognitively normal older adults, lower cerebrospinal fluid (CSF) amyloid beta 1–42 levels, indicative of abnormal amyloid aggregation in the brain, already show disrupted gray matter network measures (Tijms et al., 2016), suggesting that at very early stages of the disease networks are starting to disorganize into the direction often observed in dementia stages of AD (Tijms et al., 2013a; Pereira et al., 2016). This suggests that gray matter networks are sensitive to detect very early brain changes related to abnormal amyloid metabolism. However, as CSF is an indirect measure of amyloid plaques it remains unclear whether gray matter network disruptions are linked to local amyloid deposits or to a global effect of amyloid pathology.

In the present study, we examined the regional relationship between amyloid depositions measured with PET and gray matter network disruptions in a large cohort of cognitively normal elderly subjects with subjective memory complaints. Since the Apolipoprotein E (APOE) ε4 allele, a genetic risk factor for sporadic AD (Bertram et al., 2010), is associated with amyloid pathology (Jansen et al., 2015) and functional and structural brain changes (Cherbuin et al., 2007; Trachtenberg et al., 2012) in cognitively normal subjects we also examined whether APOE ε4 modified the relationship between amyloid and gray matter networks.

## Materials and Methods

### Subjects

We analyzed baseline data from the ongoing INSIGHT-preAD study (Dubois et al., 2018). INSIGHT-preAD is a monocentric longitudinal cohort study in 318 cognitively normal elderly (age between 70 and 85 years) with subjective memory complaints recruited from the community in the wider Paris area, France. All subjects underwent amyloid PET and MRI scans as well as an extensive battery of neuropsychological exams. Subjective memory complaints were defined by an affirmative answer to both of the following questions: “are you complaining about your memory”; “is it a regular complaint which lasts more than 6 months?”, in the absence of any objective memory deficits (mini-mental state examination (MMSE) ≥ 27, 16-item Free and Cued Selective Reminding Test (FCSRT) total score ≥ 41). Exclusion criteria were having a neurological or psychiatric disorder that could interfere with cognition (e.g., epilepsy, brain tumor, stroke), or contra-indication for MRI or amyloid PET scan. APOE genotype was determined as previously described (Teipel et al., 2017). Subjects were classified as APOE ε4 carriers if they had one or two APOE ε4 alleles and non-carrier otherwise. This study was carried out in accordance with the recommendations of the French national medical research Ethics Committee with written informed consent from all subjects. All subjects gave written informed consent in accordance with the Declaration of Helsinki. The protocol was approved by the French national medical research Ethics Committee.

### PET Acquisition and Preprocessing

Amyloid PET images were acquired on a Philips Gemini GXL CT-PET scanner using [^18^F]-Florbetapir (AVID radiopharmaceuticals). Subjects received a single intravenous dose of approximately 370 MBq (range 333–407 MBq). Fifty minutes post-injection, three 5-min frames were obtained (128 × 128 acquisition matrix, 2 × 2 × 2 mm^3^ voxels). Images were reconstructed using an iterative LOR-RALMA algorithm with 10 iterations and a smooth post-reconstruction filter. Attenuation, scatter and random coincidence corrections were integrated in the reconstruction. Frames were realigned, averaged and quality-checked. Image analysis of PET data was performed by CATI (Centre d’acquisition et traitement des images^1^). Structural MRI images were co-registered to the PET images using Statistical Parametric Mapping software version 8 (SPM8; Wellcome Department of Cognitive Neurology, London, UK). PET images were corrected for partial volume effects with the RBV-sGTM method (Thomas et al., 2011) using gray and white matter tissue maps. Using the normalization parameters from the spatial normalization of structural MRI images, a set of cortical regions of interest (ROIs) was mapped to each subjects’ native space PET. This was performed for 12 cortical ROIs (bilateral precuneus, posterior and anterior cingulate, inferior parietal, middle temporal gyrus and orbitofrontal cortex) defined in Clark et al. (2012) and a reference region (a combination of pons and whole cerebellum). For each individual, parametric PET images were created by dividing each voxel by the mean activity extracted from the reference region. Global standardized uptake value ratios (SUVr) were computed by averaging the mean activity of the 12 cortical ROIs. Regional SUVr from the 12 cortical regions was used to explore local relationships between amyloid load and gray matter networks. A global SUVr threshold for abnormality was determined by performing a linear regression analyses between the above-described method and the method used by Besson et al. (2015) which used PET scans from controls from the IMAP (Multimodal Imaging of Early-Stage AD) study. This strategy has previously been used to reliably estimate relationships between different tracers and processing methods (Landau et al., 2014). A global SUVr threshold of 0.79 corresponded to the IMAP’s cohort threshold of 1.005 (Besson et al., 2015). Thus, subjects with a SUVr above 0.79 in the present study were considered amyloid positive.

### MRI Acquisition and Preprocessing

Whole-brain scans were obtained using a 3T scanner (Siemens Magnetom Verio) with a 12-channel head coil. Isotropic structural three-dimensional T1-weighted images were acquired using a sagittal MPRAGE sequence (256 × 240 acquisition matrix, 1 × 1 × 1 mm^3^ voxels, repetition time = 2300 ms, echo time = 2.98 ms, inversion time = 900 ms, flip angle = 9°). The structural 3D T1 images were segmented using Statistical Parametric Mapping software version 12 (SPM12; Wellcome Department of Cognitive Neurology, London, UK) running in MATLAB 2011a (MathWorks Inc., Natick, MA, USA). Quality of all gray matter segmentations was visually inspected and none had to be excluded. After segmentation, all gray matter segmentations were resliced into 2 × 2 × 2 mm^3^ voxels to reduce the total number of voxels. Total gray matter volume (GMV) and total intracranial volume (TIV; i.e., GMV + white matter volume + CSF) were computed from segmented images in native space.

### Single-Subject Gray Matter Networks

Single-subject gray matter networks were computed based on cortical similarity from native space gray matter segmentations, using an automated method as previously described (Tijms et al., 2012,^2^). Briefly, nodes in these networks represent brain areas (regions of 3 × 3 × 3 voxels defined by template free approach as described in Tijms et al. (2012), and connections are based on similarity in the spatial structure of gray matter density values as quantified with a Pearson’s correlation. Networks were binarized using subject-specific thresholds as determined with a random permutation method that ensured a similar chance to include at most 5% spurious correlations in the network (Noble, 2009).

The following network measures were computed based on the average of all nodes: size of the network (i.e., total number of nodes in the network), connectivity density (i.e., ratio of existing connections to maximum possible number of connections), average degree (i.e., number of edges of a node), characteristic path length (i.e., shortest distance between two nodes), clustering coefficient (i.e., level of interconnectedness between the neighbors of a node), and betweenness centrality (i.e., the proportion of characteristic paths that run through a node). Next, we also estimated normalized path length λ and normalized clustering coefficient γ by dividing the averaged measures across nodes of each network by properties that were derived from averaging 20 randomized reference networks of equal size and degree (Maslov and Sneppen, 2002). Last, we measured the small world network property, which is defined as having more clustering than a random network while having the average path length similar to that of a random network (Watts and Strogatz, 1998). These computations were performed using scripts from the Brain Connectivity Toolbox adapted for large sized networks (Rubinov and Sporns, 2010,^3^). For regional network measures, we computed the average network properties across all nodes within each region of the automated anatomical labeling (AAL) atlas (Tzourio-Mazoyer et al., 2002). These 90 anatomical areas were defined for each subject in native space by warping the AAL atlas using the inverted parameters that were calculated when normalizing subject space images to standard space. The 12 cortical regions for which PET data was available were matched to the corresponding AAL region.

### Statistical Analysis

Demographic measures were compared between amyloid positive and amyloid negative subjects using Student’s *t*-test or Mann-Whitney-Wilcoxon test for continuous data and chi-square test for categorical data. We used two linear regression models to study the association between global amyloid burden (continuous) and each whole brain network measure. Model 1 included network measure as the dependent variable and age, gender and global amyloid SUVr as independent predictors (model 1). Additional correction for total GMV was performed in model 2. Additionally, we tested whether there was an interaction effect of APOE ε4, on the association between amyloid burden and network measures in both models.

For those network measures for which we found a global effect, we examined the regional specificity of amyloid pathology and gray matter network measures using three analyses. In the first analysis, we assessed the association between global amyloid burden and regional network measures. In the second analysis, we examined the association between regional SUVr values and network measures of the same region. In the third analysis, we used the model from the second analysis with additional correction for global SUVr. The aim of this third model was to assess whether regional SUVr values provided additional information above global SUVr. Regional associations were corrected for age, gender, TIV, local GMV and for clustering and path length also local degree. Regional associations were corrected for multiple testing using a false discovery rate (FDR) procedure (*p*_FDR_; Benjamini and Yekutieli, 2001). Regional associations were visualized using BrainNet viewer (Xia et al., 2013). All statistical analyses were performed in R (R version 3.3.1^4^).

## Results

### Cohort Characteristics

Subject characteristics for the total sample and according to amyloid status are described in Table 1. We included 318 subjects with a median age of 76 (range 69–85) and 204 (64%) were female. All subjects were cognitively normal at the time of inclusion with an average MMSE of 29 (range 27–30). All subjects had a fully connected gray matter network with an average size of 6744 nodes (SD = 619) and average network density of 15% (SD = 1). There were 88 (28%) subjects with a positive amyloid PET scan and 58 (18%) of the subjects were APOE ε4 carriers. Amyloid positive subjects were older, more often APOE ε4 carrier, had lower total GMV, lower clustering and normalized clustering γ and lower small world values. Regional amyloid PET SUVr values in the whole sample and according to amyloid status are presented in Supplementary Table S1.

**Table 1:** Clinical characteristics in total sample and according to amyloid status.

Characteristic	Total sample *N* = 318	Amyloid negative *N* = 230	Amyloid positive *N* = 88
Age years, median (IQR)	76 (74–78)	76 (73–78)	77 (75–79)**
Female, *N* (%)	204 (64%)	147 (64%)	57 (65%)
Education, median (IQR)	7 (4–8)	7 (5–8)	6 (4–8)
MMSE, median (IQR)	29 (28–29)	29 (28–30)	28 (28–29)*
FCSRT-TR, median (IQR)	47 (45–48)	47 (45–48)	46 (45–48)
APOE ε4 carrier, *N* (%)	58 (18%)	25 (11%)	33 (38%)*
PET SUVr, median (IQR)	0.71 (0.67–0.81)	0.69 (0.65–0.73)	0.97 (0.85–1.15)**
Total GMV, mean ± SD	0.567 ± 0.06	0.571 ± 0.06	0.555 ± 0.06*
Network size, mean ± SD	6744 ± 619	6759 ± 629	6703 ± 593
Network degree, mean ± SD	1036 ± 112	1040 ± 114	1026 ± 109
Connectivity density, mean ± SD	15 ± 0.8	15 ± 0.8	15 ± 0.8
Clustering, mean ± SD	0.44 ± 0.01	0.44 ± 0.01	0.43 ± 0.01*
Path length, mean ± SD	1.997 ± 0.03	1.999 ± 0.02	1.995 ± 0.03
Betweenness centrality, mean ± SD	6724 ± 610	6748 ± 626	6662 ± 564
Gamma, mean ± SD	1.54 ± 0.09	1.55 ± 0.08	1.52 ± 0.1*
Lambda, mean ± SD	1.08 ± 0.01	1.08 ± 0.01	1.08 ± 0.01
Small world, mean ± SD	1.42 ± 0.07	1.43 ± 0.06	1.41 ± 0.08*

Key: APOE, apolipoprotein E; FCSRT-TR, total recall of the Free and Cued Selective Reminding Test; GMV, gray matter volume; IQR, interquartile range; MMSE, mini-mental state examination; PET, positron emission tomography; SUVr, standardized uptake value ratio. Cut-point for amyloid positivity SUVr > 0.79. *p < 0.05, **p < 0.01 different between amyloid positive and amyloid negative subjects.

### Relationship Between Global Amyloid Burden and Whole Brain Network Measures

**Figure 1:**
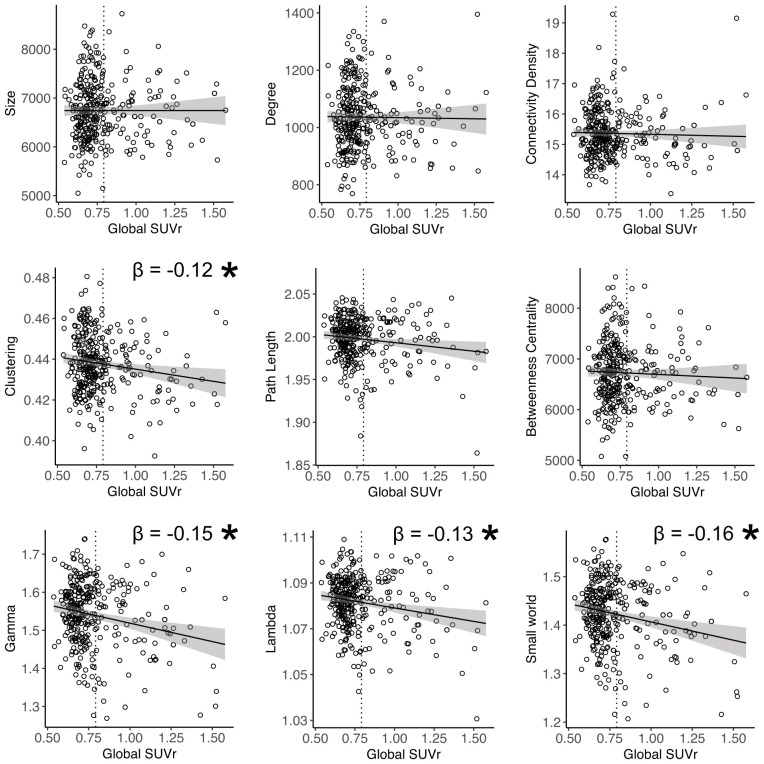
Relation between global amyloid standardized uptake value ratio (SUVr) and whole brain network measures. *Indicates significant relationship after correction for age and gender. Gamma and small world remained significant after additional correction for total gray matter volume (GMV). Dotted vertical line represents the cut-off for amyloid positivity (SUVr > 0.79).

Higher global amyloid SUVr values were associated with lower total GMV (β = −0.1, standard error 0.05, *p* = 0.04). Higher global amyloid SUVr values were associated with whole brain lower clustering, lower normalized clustering coefficient γ, lower normalized path length λ, and lower small world property when correcting for age and gender (Table 2, Figure 1). Normalized clustering coefficient γ and small world remained significant after additionally correcting for total GMV. No associations were found between global amyloid SUVr and whole brain network size, degree, network density and betweenness centrality. There was no interaction effect of APOE ε4 on the association between global amyloid SUVr and any of the network measures.

**Table 2:** Associations between global amyloid standardized uptake value ratio (SUVr) and whole brain network measures.

Network property	Model 1 β (standard error)	Model 2 β (standard error)
Gray matter volume	−0.1 (0.05)*	NA
Size	−0.03 (0.04)	0.04 (0.03)
Degree	−0.03 (0.05)	0.02 (0.04)
Connectivity density	−0.03 (0.06)	−0.03 (0.06)
Clustering	−0.12 (0.06)*	−0.1 (0.05)
Path length	−0.1 (0.05)	−0.06 (0.05)
Betweenness centrality	−0.05 (0.04)	0.02 (0.02)
Gamma	−0.15 (0.05)**	−0.09 (0.04)*
Lambda	−0.13 (0.05)*	−0.08 (0.05)
Small world	−0.16 (0.05)**	−0.09 (0.04)*

*p < 0.05, **p < 0.01. Model 1 is adjusted for age and gender. Model 2 is adjusted for age, gender and total gray matter volume. NA, not applicable.

### Relationship Between Global Amyloid Burden and Regional Network Measures

**Figure 2:**
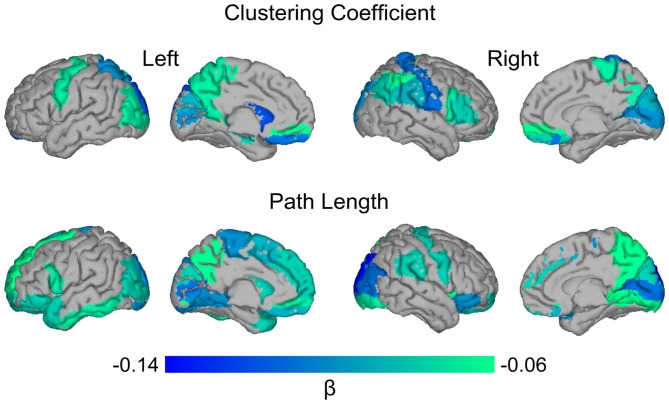
Surface plot of standardized β values of the relationship between global amyloid SUVr and local clustering and path length. Upper row: higher global SUVr was associated with lower clustering values in bilateral superior occipital gyri (left*) and gyrus rectus; left precentral, middle occipital and superior parietal gyri, precuneus, hippocampus and caudate; right superior medial orbito-frontal, inferior parietal, postcentral, supramarginal, and angular gyri, operculum, triangularis, calcarine*, cuneus, paracentral lobule and putamen. Lower row: higher global SUVr was associated with lower path length values in bilateral inferior and orbito-frontal, middle and superior occipital (right*), and lingual gyri, putamen and pallidum; left superior and medial frontal gyri, operculum, supplementary motor area, gyrus rectus, paracentral lobule, caudate, inferior temporal gyrus and middle and superior temporal pole; right precentral, precuneus, inferior occipital, and supramarginal gyri, insula, calcarine and cuneus. Data are presented for regions significant with an uncorrected *p*-value < 0.05. *Indicates region significant at *p*_FDR_ < 0.05.

Next, we examined the relationship between global amyloid burden and regional network measures to assess whether effects were localized in specific regions or equally distributed across the cortex. Higher global amyloid SUVr values were associated with lower clustering values in right calcarine and left superior occipital gyrus, and with lower path length in the right superior occipital cortex (all *p*_FDR_ < 0.05). Using a more liberal threshold of an uncorrected *p*-value < 0.05, effects were more widespread including orbito- and dorsolateral frontal and parieto-occipital cortex for clustering, and medial and orbito-frontal, posterior parieto-occipital and temporal regions for path length (Figure 2).

### Relationship Between Regional Amyloid Burden and Regional Network Measures

Subsequently we examined the relationship between regional SUVr and network measures of the same region. There were no significant associations at *p*_FDR_ < 0.05. Repeating the analysis with an exploratory uncorrected *p*-value showed that higher regional amyloid SUVr in the left precuneus was associated with lower clustering in the left precuneus (*β* = −0.06, *p* = 0.03), and higher SUVr in the right precuneus was associated with lower path length in the right precuneus (*β* = −0.08, *p* = 0.01). We also found an association between higher SUVr in right orbito-frontal cortex and lower path length in right orbito-frontal cortex (*β* = −0.06, *p* = 0.03).

**Figure 3:**
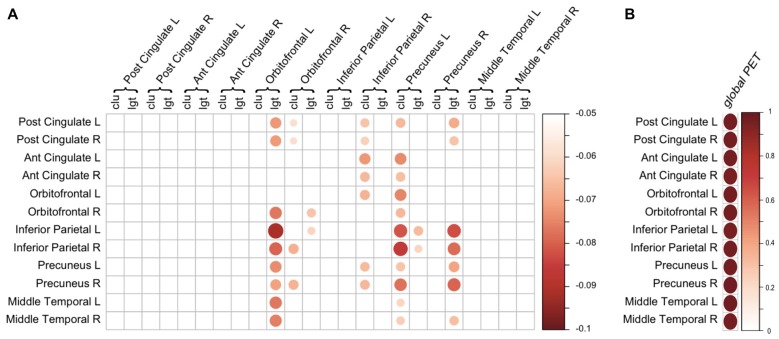
Regional associations of amyloid positron emission tomography (PET) and gray matter network measures.** (A)** Association between regional amyloid PET SUVr (rows) and regional gray matter network measures (columns). Scale indicates β correcting for age, gender, total intracranial volume (TIV) and regional GMV. Only β with a *p* < 0.05 (uncorrected) are displayed. **(B)** Correlation between regional amyloid PET SUVr (rows) and global PET SUVr (column). Scale represents the correlation coefficient. For both **(A,B)**, the size and color of the circle represent the strength of the association. Ant, anterior; clu, clustering; L, left; lgt, path length; Post, posterior; R, right.

Next, we aimed to assess whether changes in network measures were driven by regional amyloid plaques, rather than a global effect of amyloid. However, models in which we additionally corrected for global SUVr suffered from multicollinearity issues, as global SUVr was strongly correlated with regional amyloid burden (all ROIs showed a Pearson’s *r* ≥ 0.9 with a *p*-value below 1 × 10^−20^; Figure 3B). This suggests that amyloid was homogenously distributed across the cortex, which was supported by exploratory analysis that show associations of lower clustering in left precuneus with increased PET SUVr values in 10 out of 11 other regions (Figure 3A). Similarly, lower path length in right precuneus was also associated with higher amyloid SUVr values in six other regions.

## Discussion

In this study we found that increasing amyloid load measured by amyloid PET is associated with alterations in gray matter network measures in an elderly cohort of cognitively normal subjects with subjective memory complaints. Higher amyloid SUVr was associated with lower clustering, lower normalized clustering γ, lower normalized path length λ, and lower small world values. Our results suggest that gray matter network alterations may be part of the early pathological changes in AD, which can already be detected in cognitively normal subjects with subjective memory complaints in the absence of manifest cognitive impairment.

Previous studies using group level approaches have found an association between amyloid pathology and gray matter covariance in cognitively normal subjects (Oh et al., 2014; Teipel et al., 2017). Using a multivariate analysis, these studies have found amyloid pathology to be associated with a pattern of decreased GMV in medial temporal lobe, cingulate gyrus and prefrontal cortex. Using a single-subject approach to derive gray matter networks, we extend on these findings by showing within-individual associations between amyloid load and gray matter network changes.

### Relationship Between Amyloid Burden and Clustering

Results from the present study are in line with our previous study in an independent cohort of cognitively normal subjects, in which we found an association between lower amyloid beta 1–42 in CSF (representative of abnormal amyloid metabolism) and changes in gray matter network measures (Tijms et al., 2016). In that study we also found an association between increased amyloid pathology and whole brain lower clustering values, indicating that there are fewer connections between neighboring areas in the brain, suggesting less effective local integration. Here, using PET to measure amyloid depositions in the brain we extend those findings by showing that lower *normalized* clustering values γ are also associated with more severe amyloid burden. Changes in normalized clustering values γ suggest that the global network organization is also affected by amyloid deposition. In our previous study we did not find an association between normalized clustering and amyloid CSF (Tijms et al., 2016). A potential explanation for this discrepancy could be the difference in age between both populations, as subjects in the current study are approximately 20 years older than in our previous CSF study (median age 56 vs. 76 years). As amyloid pathology increases with age, subjects in the present study had on average more amyloid pathology (28% being classified as amyloid abnormal vs. 6% in the previous study). The percentage amyloid positive subjects falls within the expected range for the age group in both studies (Jansen et al., 2015). Another explanation for the differences in findings could be the method to measure amyloid pathology. Some studies have suggested that amyloid alterations may be detected somewhat earlier in CSF than on PET (Mattsson et al., 2015; Palmqvist et al., 2016), which is particularly relevant in cognitively normal subjects. CSF and PET measure slightly different aspects of amyloid pathology. In CSF, soluble amyloid beta 1–42 monomeres are measured, which decrease when amyloid aggregates in the brain. Soluble CSF amyloid beta 1–42 levels may also be influenced by other factors such as amyloid beta production and non-fibrillary aggregation (Mattsson et al., 2015), possibly making CSF more sensitive for the earliest stages of amyloid aggregation. Amyloid PET provides a more direct measure of amyloid deposition with ligands binding to the amyloid beta in fibrillary plaques (Mathis et al., 2012), leading to floor effects within the normal range. It is likely that in our previous study in a younger population that showed mostly normal CSF values, we captured the earliest signs of incipient network disorganization related to very early pathological changes. Lower clustering values associated with increased amyloid load have also been observed for structural connectivity measured with diffusion tensor imaging, independent of cognitive status (Prescott et al., 2014). Lower gray matter clustering values have previously also been reported in subjects with AD dementia and subjects with mild cognitive impairment who later convert to dementia (Tijms et al., 2013a, 2018; Pereira et al., 2016). Taking together, these studies might suggest that during the progression of Alzheimer pathology, clustering values gradually worsen starting with decreased regional connections, and progressively leading to more extensive changes rendering networks more similar to randomly organized networks.

### Relationship Between Amyloid Burden and Path Length

The relationship between amyloid pathology and path length is less straightforward. In this study we found an association between increased amyloid pathology on PET and lower normalized path length λ values, although not significant when correcting for GMV. In our earlier CSF study, we found an opposite association with lower CSF values being associated with increased un-normalized path length (Tijms et al., 2016). In that study, the increased path length values were accompanied by lower connectivity density values. With decreasing number of connections, the average path length may increase. In the present study, we did not find an association between amyloid pathology and connectivity density. Possibly, this discrepancy is explained by the age-difference between the populations studied. Network density may decrease with advancing age, and the average connectivity density was 15% in the present study, compared to 20% in our previous younger cohort. Path length values might also change non-linearly during the progression of Alzheimer pathology. Possibly, path length values first increase in the earliest stages of amyloid accumulation due to the loss of connections, and eventually decrease again when the network breaks down and becomes more randomly organized. Such an inverted U-shape trajectory of path length changes has previously been observed in functional networks during aging (Smit et al., 2012). Decreased path length values associated with network breakdown might reflect advanced disease stages when many brain areas show atrophy, and thus would show spurious similarities. In patients with AD dementia, both decreased and increased path length values have been reported across and within different imaging modalities (Xie and He, 2012; Tijms et al., 2013b; Kim et al., 2016; Duan et al., 2017). Given these inconsistencies in literature regarding path length changes in AD, and the influence of other variables on path length, path length may not be a good measure to assess and track AD-related gray matter connectivity changes. Longitudinal studies are needed to further characterize normal gray matter network changes associated with aging and pathological changes associated with amyloid pathology and brain atrophy.

### Relationship Between Amyloid Burden and Small World Values

Finally, we found an association between increased amyloid SUVr and lower small world values. Small world values indicate how much a network is locally integrated compared to a random network while remaining short path length. Small world values are based on the relation between normalized clustering coefficient and normalized path length. Hence, changes in small world values can be caused by a change in either of these measures. In this study, the decrease in small world values associated with increasing amyloid load can be explained by a relatively higher decrease in normalized clustering compared to normalized path length with increasing amyloid load. Decreases in small world values have previously also been reported in subjects with AD dementia compared to cognitively normal subjects (Tijms et al., 2013a; Kim et al., 2016; Pereira et al., 2016), and have been associated with future cognitive decline in amyloid positive non-demented subjects (Tijms et al., 2018). Some studies have also reported increased small-world values in subjects with AD dementia for different imaging modalities (Tijms et al., 2013b; Duan et al., 2017). Differences between studies might be due to differences in methods to construct the networks or non-linear changes with disease progression, possibly reflecting non-linear changes in path length. Longitudinal studies are needed to further investigate trajectories of network changes with advancing disease.

### Regional Associations Between Amyloid PET and Gray Matter Network Measures

At a local level, increased global amyloid PET SUVr values were associated with decreased clustering in orbito- and dorsolateral frontal areas as well as parieto-occipital areas. Several of the regional correlations correspond to our previous results with CSF amyloid values (Tijms et al., 2016). Increased global amyloid PET was also associated with decreased path length in various brain areas. The associations between global amyloid and regional changes were quite widespread, and some of these areas are known regions of amyloid depositions (Braak and Braak, 1996). When examining the relationship between regional amyloid load and regional network changes, we found an effect in the precuneus and orbito-frontal cortex. These may be the regions of earliest amyloid accumulation (Villeneuve et al., 2015). When we further studied the anatomical specificity of these relationships, however, we found that much of the observed associations between local network measures and amyloid pathology were largely explained by global amyloid SUVr values. Our results are in line with other studies that did not find a direct relationship between local amyloid plaque deposits and localized measures of neuronal injury (Jack et al., 2008; Altmann et al., 2015; Grothe and Teipel, 2016). Possibly, the poor anatomical correspondence between localized plaque burden and neuronal injury markers is explained by the delay in time that these biomarkers become abnormal. Amyloid pathology may start to accumulate up to 20 years before the onset of symptoms and plateaus at a relatively early stage (Jack et al., 2013; Villemagne et al., 2013). Markers of neurodegeneration on the other hand, are more closely related to the onset of symptoms (Jack et al., 2009; Da et al., 2014). Gray matter network alterations might be sensitive to detect very subtle brain structural changes associated with amyloid pathology, and precede more overt manifestations of neurodegeneration such as atrophy. Longitudinal studies are necessary to further examine the temporal relation between amyloid deposits and gray matter network changes. Possibly, the observed association between amyloid and gray matter network measures may reflect the presence of tau in addition to amyloid pathology. Regional tau deposits may show more clear associations with regional disruptions of brain structure and function (Ossenkoppele et al., 2016; Xia et al., 2017). With the advent of new tau-binding ligands for PET, the anatomical relation between amyloid plaques, tau deposits and gray matter network changes can be examined in future studies (Villemagne et al., 2015).

### Effect of APOE

In agreement with previous studies in cognitively normal subjects, we did not find an effect of APOE ε4 genotype, a major genetic risk factor for AD, on the association between amyloid pathology and gray matter network measures (Oh et al., 2011; Tijms et al., 2016; Teipel et al., 2017). Although APOE ε4 genotype has been associated with amyloid pathology in cognitively normal subjects in a large meta-analysis study (Jansen et al., 2015), it seems that subsequent structural brain alterations are not different for APOE ε4 carriers and non-carriers. This suggests that APOE ε4 most strongly affects (the age of) amyloid aggregation, but not necessarily the anatomical locations that will show most pronounced structural brain changes.

### Limitations

A potential limitation of the present study is that we only had local SUVr values available for a subset of anatomically relevant cortical regions, for which the regional SUVr were all highly correlated with global SUVr. As such, the possibility that other anatomical areas might show more variability in amyloid depositions cannot be excluded (Villain et al., 2012). Additionally, amyloid load was assessed using semiquantitative SUVr values, which do not take into account confounding variables that may influence tracer uptake, such as flow effects, and so this might have introduced noise to the data (van Berckel et al., 2013). We presently studied subjects with subjective memory complaints, a population that might be enriched for preclinical AD, because these subjects may have higher chances of amyloid pathology and be at increased risk of cognitive decline (Jessen et al., 2014). Although this makes our study clinically relevant, this limits generalizability to the broader population. We used a cross-sectional approach to study the relationship between amyloid PET and gray matter networks. Longitudinal amyloid PET and structural MRI data might give more insight into the relationship between amyloid pathology, gray matter network disruptions and cognitive decline. Finally, it is possible that the association between amyloid and gray matter network changes reflects the presence of tau pathology. We were not able to examine this in the present sample as we did not have information on tau pathology from CSF or PET. Future studies may focus on examining the relationship between amyloid, tau and gray matter network changes.

## Conclusion

In summary, we found that in cognitively normal subjects, global amyloid burden is associated with alterations in gray matter network measures. These results suggest that gray matter network alterations may occur at a very early stage in the pathogenesis of AD.

## Author Contributions

MK and BMT analyzed the data and drafted the manuscript. PJV, HB, FB, SAMS, WMF, PS, HH, M-OH and BD revised the manuscript for important intellectual content. HB, HH, BD and BMT conceived and designed the study.

## Conflict of Interest Statement

The authors declare that the research was conducted in the absence of any commercial or financial relationships that could be construed as a potential conflict of interest.

## Footnotes

^1^cati-neuroimaging.com

^2^https://github.com/bettytijms/Single_Subject_Grey_Matter_Networks

^3^www.brain-connectivity-toolbox.net

^4^http://www.R-project.org
