# Supplementary material for: Gray Matter Network Disruptions and Regional Amyloid Beta in Cognitively Normal Adults
Source: Front Aging Neurosci. 2018 Mar 15;10:67. doi: 10.3389/fnagi.2018.00067 (PMC5863592; doi:10.3389/fnagi.2018.00067)
Supplement: Supplementary file 1 [file Table_1.PDF]

SUPPLEMENTARY MATERIAL

Gray matter network disruptions and regional amyloid beta in cognitively normal adults

Mara ten Kate, Pieter Jelle Visser, Hovagim Bakardjian, Frederik Barkhof, Sietske A.M. Sikkes, Wiesje M. van der Flier, Philip Scheltens, Harald Hampel, Marie-Odile Habert, Bruno Dubois, Betty M. Tijms
for the INSIGHT-preAD study group

Supplementary table I: Regional amyloid PET SUVr values in total sample and according to amyloid status

Region	Total sample<br>N = 318	Amyloid negative<br>N = 230	Amyloid positive<br>N = 88
Left posterior cingulate	0.67 (0.61-0.76)	0.64 (0.59-0.68)	0.95 (0.83-1.12) *
Right posterior cingulate	0.65 (0.60-0.77)	0.63 (0.59-0.67)	0.94 (0.81-1.11) *
Left anterior cingulate	0.65 (0.60-0.76)	0.62 (0.58-0.66)	0.93 (0.78-1.06) *
Right anterior cingulate	0.68 (0.63-0.79)	0.66 (0.61-0.69)	0.98 (0.84-1.13) *
Left orbito-frontal cortex	0.82 (0.76-0.93)	0.79 (0.74-0.84)	1.08 (0.96-1.24) *
Right orbito-frontal cortex	0.79 (0.73-0.90)	0.76 (0.71-0.81)	1.06 (0.94-1.22) *
Left inferior parietal cortex	0.73 (0.66-0.84)	0.69 (0.64-0.74)	0.98 (0.86-1.12) *
Right inferior parietal cortex	0.72 (0.66-0.83)	0.68 (0.64-0.73)	0.97 (0.88-1.16) *
Left precuneus	0.69 (0.64-0.79)	0.66 (0.61-0.70)	0.98 (0.84-1.14) *
Right precuneus	0.69 (0.63-0.79)	0.65 (0.62-0.70)	0.96 (0.84-1.16) *
Left middle temporal gyrus	0.75 (0.70-0.84)	0.73 (0.68-0.76)	0.97 (0.88-1.16) *
Right middle temporal gyrus	0.77 (0.71-0.86)	0.74 (0.69-0.78)	0.98 (0.89-1.22) *

Data are presented as median (IQR).
Key: IQR, interquartile range; PET, positron emission tomography; SUVr, standardized uptake value ratio. * $p < 0.001$ different between amyloid positive and amyloid negative subjects.
